# Mechanisms of Myocardial Edema Development in CVD Pathophysiology

**DOI:** 10.3390/biomedicines12020465

**Published:** 2024-02-19

**Authors:** Diana G. Kiseleva, Tatiana V. Kirichenko, Yuliya V. Markina, Vadim R. Cherednichenko, Ekaterina A. Gugueva, Alexander M. Markin

**Affiliations:** 1 Department of Biophysics, Faculty of Biology, Lomonosov Moscow State University, 119991 Moscow, Russia; 2 Laboratory of Cellular and Molecular Pathology of Cardiovascular System, Petrovsky National Research Centre of Surgery, 119991 Moscow, Russiacherednichenko_vadim@bk.ru (V.R.C.);; 3 Chazov National Medical Research Center of Cardiology, Ac. Chazov Str. 15A, 121552 Moscow, Russia; 4 N.V. Sklifosovsky Institute of Clinical Medicine, I.M. Sechenov First Moscow State Medical University, 119435 Moscow, Russia; katya.guguewa@yandex.ru; 5 Medical Institute, Peoples’ Friendship University of Russia Named after Patrice Lumumba (RUDN University), 117198 Moscow, Russia

**Keywords:** myocardial edema, cardiomyocytes, cardiovascular diseases, myocardial fluid flow

## Abstract

Myocardial edema is the excess accumulation of fluid in the myocardial interstitium or cardiac cells that develops due to changes in capillary permeability, loss of glycocalyx charge, imbalance in lymphatic drainage, or a combination of these factors. Today it is believed that this condition is not only a complication of cardiovascular diseases, but in itself causes aggravation of the disease and increases the risks of adverse outcomes. The study of molecular, genetic, and mechanical changes in the myocardium during edema may contribute to the development of new approaches to the diagnosis and treatment of this condition. This review was conducted to describe the main mechanisms of myocardial edema development at the molecular and cellular levels and to identify promising targets for the regulation of this condition based on articles cited in Pubmed up to January 2024.

## 1. Introduction

Excessive fluid accumulation in the myocardial interstitium or in the cardiac cells may develop due to changes in capillary permeability, loss of charge of the glycocalyx, imbalance in the lymphatic system’s drainage, or a combination of these factors [1,2]. This condition is called myocardial edema. Although it was previously believed that myocardial edema was a secondary phenomenon and a consequence of acute and chronic cardiovascular diseases (CVDs), it has recently been shown that edema itself causes aggravation of the disease, increasing the risks of adverse outcomes, and often leads to irreversible changes in the interstitium structure [3]. For example, excess fluid in the myocardium can lead to fibrosis through stimulation of increased collagen synthesis and its increased deposition in the interstitium due to the activation of TGF-β and procollagen types I and III [2]. It has also been shown that cardiac muscle dysfunction can be directly related to edema without associated damage, which only emphasizes the importance of the development of this pathological condition [4].

Currently, the etiology of myocardial edema, its relationship with the excitation–contraction coupling, as well as the regulation of fluid flow between the capillary and cardiomyocytes generating pressure > 100 mmHg every second [5], and the lymphatic system is not completely understood. Myocardial edema can be observed in coronary heart disease due to hypoxia [6], myocardial infarction [7,8], myocarditis [9], and various types of cardiomyopathies [1], which account for more than 70% of all deaths from cardiovascular diseases in the world [1,10]. In addition, the edema is found in such chronic conditions as arterial and pulmonary hypertension, as well as during the usage of cardiopulmonary bypass, cardioplegic arrest [11], and sepsis [1].

Myocardial edema impairs the contractility of cardiomyocytes and is the cause of systolic and diastolic dysfunction. Numerous experimental data indicate that with an increase in fluid content in the myocardium of just a few percent, cardiac output can easily decrease by 15 percent and more [2,12,13]. A murine model with myocardial edema caused by elevated coronary sinus pressure demonstrated contractile dysfunction within 24 h and subsequent development of inflammation and fibrosis in the left ventricle [2,13].

Thus, myocardial edema covers a colossal amalgam of cardiovascular diseases, not only being a consequence of various disorders, but also independently leading to complications and increasing the risk of complications. Further research in this field and practical application of the results obtained in the studies could help to shed light on many issues of molecular, genetic, and mechanical changes in the myocardium during edema, which could potentially help alleviate acute conditions for patients. The purpose of this review is to focus on myocardial edema at the cellular level and assess the prospects for development in this direction.

## 2. The Fluid Flow in Myocardial Tissue under Normal Conditions

The myocardium consists not only of contractile cells of the heart, cardiomyocytes, but also fibroblasts, which form the rigidity of the interstitial space and the distribution of cardiomyocytes along the axes, blood capillaries surrounded by pericytes, and lymphatic capillaries. The extracellular space also contains fat, interstitial pores, and cells of the immune system. Moreover, cardiomyocytes are connected by gap junctions, not only to each other but also to fibroblasts, which also contribute to the excitation wave propagation [14]. Normally, the regulation of fluid flow in the myocardium occurs due to the balance of osmotic and oncotic pressure. Under pathophysiological conditions, large amounts of proteins accumulate in the interstitial space, creating an osmotic/oncotic pressure force that leads to fluid flow into the tissue and prevents fluid from being reabsorbed into the bloodstream. This chain of events creates the necessary conditions for the formation of edema [2].

The rate of fluid filtration through the microvessel wall is described using the Starling–Landis equation [15,16]:

(1) $$J_{v}=K_{f}\left[\left(P_{c}-P_{i}\right)-\sigma \left(\Pi_{c}-\Pi_{i}\right)\right],$$

where *P_c_* is the end-diastole microvascular hydrostatic pressure, *P_i_* is the end-diastole interstitial hydrostatic pressure, *Π_c_* is the plasma osmotic pressure, and *Π_i_* is the interstitial osmotic pressure. The difference between hydrostatic or osmotic pressure causes fluid to enter the interstitium and to retain or draw fluid in the opposite direction from the interstitium to the capillaries. *K_f_* represents the microvascular filtration coefficient and *σ* is the reflection coefficient, which characterizes the relative permeability of the capillaries towards plasma proteins.

**Figure 1:**
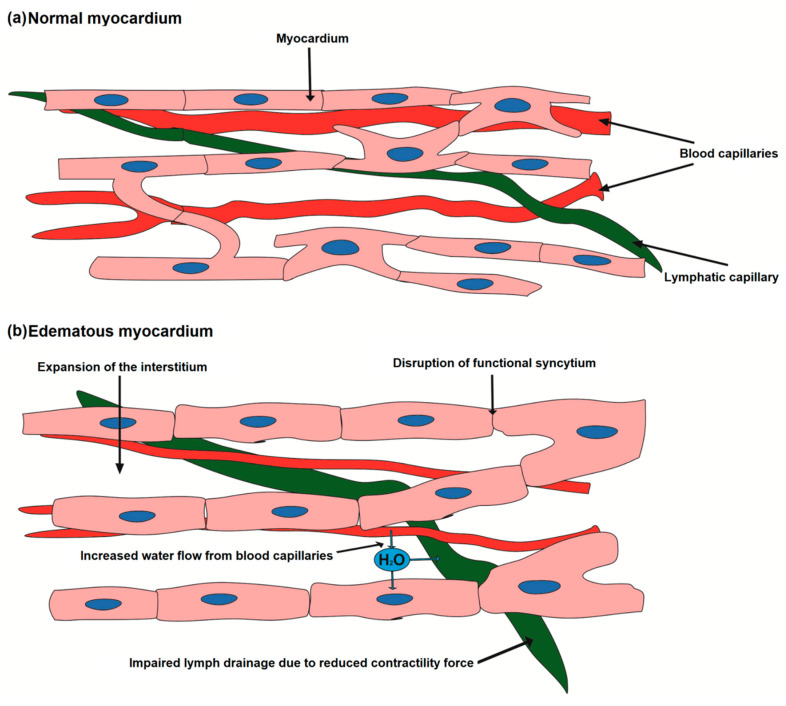
Imbalance of fluid flow in the myocardium and the development of edema. Normally, there is a constant flow of fluid from the vessels in the interstitium and from the interstitium into the lymphatic vessel. Myocardial contractions help to stimulate cardiac lymphatic flow. When the flow from the vessel is higher than the filtration capacity of the lymphatic capillaries, swelling occurs.

Lymphatic vessels are involved in the transport of excess fluid from the interstitium, but for interstitial drainage and lymph transport the normal pumping function of the heart within certain limits is required [17], since small lymphatic capillaries do not have contractile cells [18,19]. In order to push lymph, the pressure created by cardiomyocytes during systole is necessary [20,21]. Hence, pulsating shocks of blood pressure into the capillaries can lead to the penetration of fluid into the interstitial space under normal conditions. The change in osmotic pressure in the interstitium generates a pressure difference between the lymphatic vessels and the interstitium, resulting in fluid flow into the lymphatic vessel [22,23,24]. Systolic contraction of the myocardium pushes excess fluid further through the lymphatic network. An imbalance in this process can lead to fluid stagnation in the interstitium and/or cells, only worsening the edema and leading to a vicious circle. Figure 1 demonstrates the pathogenesis of the myocardial edema.

Dongaonkar et al. analyzed the physiological ranges for the variables in this equation based on experimental data [11]. *K_f_* depends on the surface area of the myocardial capillaries and hydraulic conductivity. At the same time, the density of the microvascular network in the myocardium is very high (3000–4000/mm^2^) [25], with the capillaries being very close to the cardiac cells. This density is necessary to ensure an optimal oxygen diffusion distance and access to nutrients. Accordingly, the total surface area of the capillaries is greater than in other organs, increasing *K_f_*. An indirect estimate of myocardial *K_f_* is ~0.35 mL min^−1^ mmHg^−1^ 100 g^−1^ [11]. Bravo-Reyna et al. measured *K_f_* in rats using two different techniques and obtained similar results for both of them with *K_f_* of 0.21–0.28 mL min^−1^ mmHg^−1^ 100 g^−1^ [26]. In comparison, for swelling human skeletal muscles this coefficient was measured as 3.2–5 × 10^–3^ mL min^−1^ mmHg^−1^ 100 g^−1^ [27]. Hydrostatic pressure in capillaries (*P_c_*), according to experimental data, is approximately 20–30 mmHg.

Measuring *Π_c_*, the osmotic pressure created by plasma proteins, whose molecular weight is >30,000 Da, under native conditions is quite difficult. It is directly measured through an artificial membrane, which does not accurately reproduce the capillary membrane. However, the measured values reported in the study are 21–24 mmHg for humans and 17–19 mmHg in dogs. Plasma osmotic pressure, *Π_i_*, cannot be measured directly, but is thought to be similar to that measured in myocardial lymphatics (14 mmHg for dogs) [11].

The model itself does not consider the role of myocardial cells and their hydraulic conductivity. The hydraulic conductivity regulation of the cardiomyocyte membrane occurs due to aquaporin types 1 and 4, Na^+^/HCO^3−^ symporter, Na^+^/Ca^2+^ exchanger, Na^+^/H^+^ exchanger [18], as well as the sodium and chloride channels’ role, was emphasized by early experimental studies [28,29]. Aquaporins 1 are localized in the t-tubule region, playing a role during osmotic stress, including ischemia/reperfusion and cardiopulmonary bypass [30].

The accumulation of excess fluid inside the cell triggers depolymerization of actin filaments, mitochondrial damage, cytoskeletal abnormalities, and a significant increase in the length of sarcomeres, the radial distance between myofibrils and the distance between mitochondria and myofibrils, which directly affects the contractility of cells [25]. Myofibrils make up 45 to 60% of the volume of cardiomyocytes [31], while mitochondria make up about 40% [32]. T-tubules limit the diffusion of extracellular fluid by creating a microdomain of ions with a relatively stable concentration compared to the wider extracellular space. Such mechanisms may prevent the negative effects of rapid changes in extracellular fluid [33] and the cardiomyocyte morphological structure may be the reason for the slower cell swelling under hypo-osmotic conditions.

Excessive mechanical stress, including interstitial and cellular edema, contributes to changes in mitochondrial homeostasis, leading to cardiac dysfunction [34,35]. Mechanical stress disrupts the interaction of cardiomyocytes with the extracellular matrix (ECM), which provides the microenvironment for myocardiogenesis and maturation [32]. ECM consists of fibronectin, glycoproteins, proteoglycans, and glycosaminoglycans and contains cytokines including TGF-β, BMP, platelet-derived growth factor, and connective tissue growth factor [36,37]. The increase in force exerted by the extracellular matrix induces a wide range of intracellular signals, including signals from the Akt, c-Jun N-terminal kinase, and MAPK-ERK-p38 pathways. Ras-like small GTP-binding proteins are major components of myocardial biochemical sensors and are involved in cardiovascular diseases, for example, activation of the RAS/RAF-dependent MAPK signaling induces hypertrophic cardiomyopathy [38]. Moreover, a large number of proteins of the Ras superfamily are involved in the regulation of Ca^2+^ channels, mediating the excitation–contraction coupling and heart rate [32]. The molecular pathways that are activated during edema have not been studied, but, most certainly, severe mechanical stress will influence the restructuring of the cardiomyocyte microenvironment and impair cell contractility, which only emphasizes the importance of further research.

## 3. The Role of Gap Junctions in Myocardial Edema

Cardiac tissue can be regarded as a functional syncytium due to the connections of cardiac cells in the areas of intercalated discs (IDs) by ion channels, cadherins, and gap junctions. However, not all gap junctions are functionally present in the ID, typically only up to 20% [39]. Processes that regulate gap junction conductance, tissue geometry, and varying degrees of connections between cells result in nonlinear regulation of ionic currents that change membrane potential and calcium wave propagation, which induces contraction. Such a high level of anisotropy could partially explain the anomalous diffusion phenomena observed in experiments [14,40].

In the cardiovascular system, various connexins are expressed, including connexin Cx26, Cx30, (Cx)31.9, Cx32, Cx37, Cx40, Cx43, Cx45, Cx46, and Cx50 [39,40]. Cardiomyocytes express three main connexin isoforms, Cx43, Cx40, and Cx45; however, the most common, Cx43, is the main connexin isoform expressed in the working ventricular and atrial myocardium [39]. One gap junction channel is formed by docking two half-channels (connexons), each of which consists of six connexin molecules arranged hexagonally around an aqueous pore. Gap junction channels are permeable to substances with a molecular mass of <≈1 kDa. The permeability depends on the type of connexin and the charge of the penetrating molecule [41]. Cx26, Cx30, Cx37, Cx40, Cx46, and Cx50 close under positive voltages, whereas Cx31, Cx31.9 (mCx30.2), Cx32, Cx43, Cx45, and Cx57 close under negative voltages [42].

**Figure 2:**
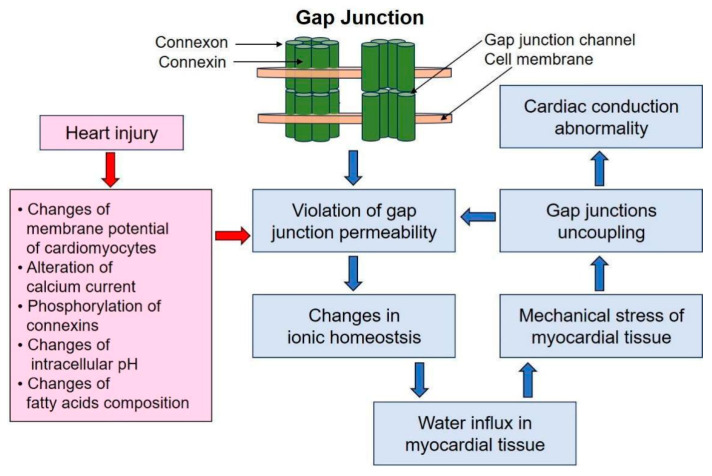
The role of gap junctions in the pathogenesis of myocardial edema.

Gap junction conductance is regulated by membrane potential, proton and calcium currents, the phosphorylation state of connexins, and extracellular fatty acid composition [41]. Connexin expression is also modulated by changes in these factors. Changes in membrane potential can occur in certain pathological situations, such as myocardial ischemia, especially in the border zone surrounding the ischemic area. These changes may lead to gap junction channels’ closure, thereby electrically isolating the area from the rest of the myocardium. This helps to prevent the abnormal action potential propagation; however, at the same time, electrical isolation can lead to areas of conduction blockage [39]. In addition to intercellular communication, gap junction connexins play an important role in maintaining cellular homeostasis; under pathological conditions, this causes disturbances in ion transport [43] that might lead to intracellular edema of cardiomyocytes. In turn, myocardial edema has the potential to affect the function of gap junctions. Edema can cause mechanical stress of the heart tissue, leading to disruption of gap junctions’ integrity and function, with the subsequent impairment of the electrical coupling between cardiomyocytes that underlies the development of myocardial conduction abnormality [44]. The role of gap junctions in the pathogenesis of myocardial edema is presented in Figure 2.

Many studies have confirmed the modulation of gap junction conductance by intracellular pH; with cytoplasmic acidification, which is also observed during ischemia, a decrease in gap junction conductance occurs [39]. A number of studies showed that in ischemic and hypertrophied human left ventricles there was a reduction of up to 40% in the number of gap junction clusters, but the number of IDs per cell did not differ in normal and diseased hearts, meaning that cell geometry did not change dramatically. Similar results were obtained on cardiomyocytes of guinea pigs and rats [41]. A strong correlation has also been demonstrated between the induction of figure-of-eight recurrent arrhythmias in epicardial tissue adjacent to 4-day infarcts in canine ventricles and disruption of gap junction distribution [42]. In addition, an age-dependent decrease in Cx43 is associated with a slower conduction velocity and an increased risk of arrhythmias [45].

Although there is evidence that gap junction lateralization characterizes a diseased myocardium, it is not entirely clear to what extent this lateralization may contribute to changes in excitation propagation properties. It was recently shown that in rat ventricular cells near healed infarcted areas, many of the lateral gap junction clusters are located in sarcolemmal invaginations within the cell, thereby not facilitating intercellular communication. A similar observation was made in a right ventricular hypertrophy model [41]. The relationship between cardiac gap junction conduction, cell volume, and susceptibility to arrhythmias was studied by Veeraraghavan et al. In their research, the authors analyzed the extent to which interstitial volume modulates conduction velocity and its dependence on cell connectivity. Interstitial volume was determined histologically in the right ventricle of the guinea pig. Optical mapping was used to quantify conduction velocity and anisotropy. Their results showed that changes in interstitial volume modulate conduction velocity, anisotropy, and the conductance-to-connectivity ratio of cells and thereby alter susceptibility to arrhythmias [46]. Changes in osmolality will significantly affect the above parameters.

Interestingly, the ambiguous relationship between conductance and connexin expression in pathological conditions may be due to the fact that gap junctions promote changes in ionic homeostasis within cells and water influx in myocardial tissue. In HeLa cells, alkalization of the extracellular environment leads to calcium overload and is dependent on the presence of active Cx43 hemichannels [47]. Furthermore, ion entrance to the cytosol is passively followed by water influx, leading to cell edema. In Xenopus oocytes it was found that overexpression of Cx46 depolarizes and lyses cells within 24 h and is associated with the appearance of voltage-gated currents in non-junctional membranes and water influx [39]. A blocker such as Gap26, which blocks Cx43 in cardiomyocytes and fibroblasts, reduces infarct size following ischemia–reperfusion and improves cardiomyocytes’ viability. At the same time, rotigaptide, which acts on cardiomyocytes and endothelial cells, by facilitating Cx43 gap junctional coupling, reduces infarct size and endothelial dysfunction [42].

## 4. Cellular Models of Myocardial Edema

Studies on cardiomyocytes edema began relatively recently; the first most detailed studies on cells were carried out in the 1990s–2000s. Basically, edema is simulated by placing cells in solutions with altered osmolality. The osmolality is adjusted by varying the glucose content, ionic composition, in particular sodium content, or by diluting the isosmotic solution with distilled water. Selecting solutions with different sodium osmolality is associated with the most common electrolyte imbalance in patients, which leads to varying symptoms depending on severity, and is also associated with higher mortality from CVD [48,49]. Differences in osmotic and oncotic pressure locally in the myocardium can occur not only due to sodium, for example, but due to lactate during ischemia; hence, modeling of the ionic imbalance between the interstitium and the intracellular space is even more complicated. However, such a solution content may be driven by the fact that the change in osmotic pressure in the interstitium occurs mainly due to pathologically increased capillary permeability [12].

The earlier experiments were aimed at studying single isolated cells and membrane channels for volume regulation, in particular due to Cl^−^ current, as well as aquaporins. Changes in cell volume and swelling rate were analyzed by processing microscopic images and videos. For example, volume changes induced by the osmotic gradient and cardiomyocyte hydraulic conductivity were measured in isolated guinea pig and rat ventricular cardiomyocytes using digital video microscopy, and membrane hydraulic conductance was determined by analyzing the dynamics of cell swelling in response to osmotic gradients. The results of the study showed that changes in osmolality caused rapid (less than 3 min to steady state) and reversible swelling or contraction of cardiomyocytes [29]. It was also shown in guinea pigs that chloride channels are activated during cell swelling and are involved in the process of volume regulation, and also depend on the membrane potential and the equilibrium Cl^−^ potential [28]. Osmotic regulation of cell volume influences a number of other sarcolemmal currents, including K^+^ channel activity, which has a significant impact on cardiac electrical activity and susceptibility to arrhythmias [12].

With the development of research methods, morphological changes in cardiomyocytes during edema were studied. The edema was found to be associated with subsarcolemmal blebs. Exposure to a hypotonic environment caused sarcolemmal rupture in metabolically inhibited adult rat cardiomyocytes. It was concluded that osmotic stress induces sarcolemmal breakdown and that this effect is not due to the low concentration of Na^+^ present in the hypo-osmotic buffer, and the osmotic fragility of the sarcolemmal blebs persists for at least 40 min after restoration of metabolic activity [50].

In isolated control and ischemia-induced rabbit cardiomyocytes, the formation of blebs was detected by 75 min following acute hypo-osmotic edema. Osmotic fragility developed only after 75 min [51]. Human and rat cardiomyocytes have also been studied by scanning ion-conducting microscopy; Lyon et al. discovered loss of t-tubules in heart failure [52], which is accompanied by edema. Over the past 30 years it was also found that swelling or shrinkage of cardiomyocytes alters the duration of the action potential, causing a marked loss or increase in the plateau phase of the action potential in guinea pig and cat models [53,54].

At the same time, experimental data on mechanistic changes, i.e., the cardiomyocytes’ volume and their shape, indicate that the change in cells is not proportionate, mainly occurring in the transverse direction rather than in the longitudinal direction. When cardiomyocytes were exposed to a solution containing 60% sodium ions compared to isosmotic conditions, the width increased up to 118% of the control, while the length increased only up to 104%, which emphasizes the importance of the geometrical features and the difference in elastic moduli [55]. An in vivo study of the effect of acute changes in osmolality and sodium concentration on myocardial contractility was conducted in dogs. It was found that the sodium concentration and osmolality had independent effects on myocardial contractility, reducing it. Hyponatremia caused a decrease in contraction by 17 ± 4% (*p* < 0.01) [56].

Indeed, the spread of edema and its effects differ significantly between isolated cells and cell monolayers. Very few experiments have been performed on a monolayer, let alone on a 3D cell culture [57]. Thus, cardiac excitation propagation through gap junctions is an important factor determining susceptibility to arrhythmias. The relationship between the degree of uncoupling (the number of intercellular contacts) and conduction velocity was revealed using optical mapping in the right ventricle of a guinea pig [46]. An assessment of the contribution of gap junctions with increasing fluid volume in cells was shown using the example of a spheroid from the normal epithelial cell line MCF10A; the results on cells corresponded to the linear model proposed by the authors [58].

Thus, 3D tissue architecture is of particular interest in the study of edema, since cell geometry and microenvironmental stiffness may influence the response of cardiomyocytes to anisosmolar solutions. Cell morphology can change significantly depending on the stiffness of the substrate, and it can also influence molecular crowding, even under isotonic conditions. Using cell lines A7, HeLa, NIH 3T3, mouse mesenchymal stem cells, and primary human airway smooth muscle cells, it was shown that on the stiffest substrates the volume reduction reaches ~40% compared to cells grown on the softer substrates, where the cell volume is a maximum [59].

## 5. Diagnostic Methods of Myocardial Edema and Potential Treatment

### 5.1. Diagnostic Methods

Myocardial edema is an important prognostic factor in CVD [60]. Its severity and localization may indicate the possible further development of complications. The accumulation of fluid in the myocardium leads to systolic and diastolic ventricular dysfunction. It is not possible to identify any specific signs of myocardial edema on an ECG. However, myocardial edema can be assumed by the presence of local thickening of the ventricular wall [14,61,62]. The most informative method is histological examination, but it is not possible to take a biopsy from every patient. Previously, myocardial edema was quantified using gravimetric methods. With this method, the water content of the myocardium is determined by measuring the specific density of small myocardial samples of about 5 mm^3^ using a linear density gradient. Knowing the specific gravity of a myocardial sample, the percentage of water per gram of tissue can be calculated. However, due to its invasive nature, the clinical application of this method is limited [1].

Myocardial edema is clearly visualized on cardiac MRI [61,63]. For MRI, the signal emanating from hydrogen atoms is most often used, as they are most represented in biological tissues. This diagnostic method is based on the ability of MR-active atomic nuclei, which have their own charge and spin, acquire a magnetic moment and can be oriented in an external magnetic field (B_0_), and absorb energy when exposed to an RF band close to the frequency of its own oscillations, entering into resonance. Most of the magnetic moments of such nuclei are oriented parallel to B_0_, providing a net magnetic vector (NMV) sufficient to form an adequate image of organs and tissues. Although, when using radio frequency coils tuned to the appropriate frequency, it is possible to receive a signal from other MR-active nuclei oriented antiparallel [64].

So, receiving energy, the nucleus resonates; resonance leads to the fact that NMV is located not parallel to the B_0_ direction, but at a certain angle to it. This angle is called the flip angle, and its magnitude depends on the amplitude and duration of the RF pulse. For example, if the RF pulse positioned the NMV at 90° relative to B_0_, then B_0_ is called the longitudinal plane, and the plane located at an angle of 90° relative to B_0_ is called the transverse plane. At this stage, a potential difference will arise, which represents the MR signal. During relaxation, the nuclei lose the absorbed RF energy and the NMV returns to B_0_. Relaxation leads to restoration of magnetization in the longitudinal plane and to its decline in the transverse plane. The restoration of magnetization in the longitudinal plane is called T1 recovery, and the decrease in magnetization in the transverse plane T2 relaxation. The image contrast can be adjusted through various parameters, including T1 or T2 weighting, which depend primarily on the time differences for each type of relaxation between fat and water [64].

On a T1-weighted heart MRI, scar changes in myocardial tissue, namely, fibrosis, are clearly visualized, on the other hand, the T2 relaxation time increases with excessive water content in the myocardium. Therefore, on a T2-weighted image, myocardial edema is detected as an area of high signal intensity [63]. There are different types of RF pulse sequences for T2-weighted images. The turbo spin echo (TSE) pulse sequence with dark blood is most commonly used to visualize edema. TSE involves first delivering an RF pulse at 90°, followed by a series of multiple pulses at 180° [65]. An effective sequence is also the triple inversion turbo spin (T2-STIR). After myocardial infarction (MI), the area of edema may identify an area of increased risk for recurrent infarction. T2-weighted imaging allows one to distinguish between edema in acute and chronic myocardial ischemia [61]. Myocardial edema can also be visualized by injecting a contrast agent, such as gadolinium. Edema is characterized by the so-called phenomenon of “late gadolinium enhancement”—a delay in the removal of contrast at 10–15 min of the study when high-intensity signal zones appear [66,67].

The use of cardiac MRI sequences allow the differentiation of a potentially reversible acute heart injury and a chronic irreversible myocardial lesion that has a high diagnostic and prognostic value in some cardiac diseases, such as acute coronary syndrome or myocarditis [68]. In acute myocardial infarction, T1 sequences with gadolinium contrast reveal the infarct size while T2 sequences allow identification of myocardial edema as a hyperintense zone of viable myocardium; so, the use of MRI has not only diagnostic value, but can also be used for monitoring of the treatment effectiveness [69]. In turn, in acute myocarditis, the presence of edema on MRI, identified as a subepicardial or intramyocardial increased signal intensity on T2 images, is associated with a favorable prognosis [70,71].

The localization of edema in the myocardium correlates with its nature. Thus, in MRI, edema caused by ischemia is detected along the coronary artery and often has a transmural localization. In cardiomyopathy and myocarditis, edema is usually located in the middle layers of the myocardium and in the subepicardial layers and is not localized along the coronary artery [72,73,74]. Moreover, if the area of edema is smaller than the area of scarring, then this is the acute phase of MI. However, T2-weighted imaging has several limitations. Various artifacts are often found in the image, which reduces the accuracy of this method [41]. A more effective and sensitive diagnostic method in detecting diffuse edema and minimal changes in T2 relaxation values is T2 mapping [61,63].

T2 mapping represents an image of the myocardium displayed pixel-by-pixel at several time points, reproducing the T2 relaxation curve for each pixel. As a result, a color image is obtained that allows edema to be visualized and quantified by relating the normal intensity in patients without edema to the area of increased intensity during mapping. A series of images is taken during a breath hold and several RR intervals on the cardiogram. T2 mapping can also use a variety of sequences, including turbo spin echo, multispin echo, gradient echo, or balanced steady-state free precession (bSSFP). Mapping helps to reliably diagnose edema of ischemic nature, as well as myocardial edema of an infectious nature associated with cardiomyopathy and transplantation [75]. Furthermore, T2 mapping makes it possible to establish that severe myocardial edema accompanies light-chain amyloidosis (AL). Compared with transthyretin amyloidosis (ATTR), AL amyloidosis has a worse prognosis and higher mortality, which is associated with more severe edema [76].

Other methods have been proposed to determine myocardial edema, for example, speckle-tracking echocardiography [77]; several studies have found associations between the conventional ECG method and myocardial edema, but the accuracy of such a diagnosis is controversial. Studies also suggest that T-wave abnormalities associated with non-ST-segment elevation acute coronary syndrome may be associated with the presence of myocardial edema and indicate a potentially reversible change in ischemic myocardium associated with worse outcomes [78].

Another complex problem of diagnostic methods concerns distinguishing intracellular and extracellular myocardial edema. Despite attempts to develop a method for differentiating intracellular excess myocardial fluid from extracellular fluid [79], reliable results that can be applied in practice have not been obtained. Further research in this area may allow a better understanding of the mechanisms and consequences of myocardial edema and, as a result, lead to new diagnostic and therapeutic applications [80].

### 5.2. Myocardial Edema Treatment

Various pathological conditions associated with excessive accumulation of fluid in different organs significantly worsen the condition of patients and require effective treatment. Many heart diseases are also associated with the development of edema not only in the myocardium, for example, heart failure leads to stagnation of fluid in the systemic or pulmonary circulation (or both), as a result, the function of many vital organs is impaired This condition is quite effectively eliminated by diuretics. For example, the loop diuretic furosemide or the potassium-sparing diuretic spironolactone [81]. However, myocardial edema is a local accumulation of fluid that has different locations, degrees, and primal causes, and a more effective therapy than diuretics is required.

#### 5.2.1. Therapy Aimed at Stimulating Lymphangiogenesis

**Figure 3:**
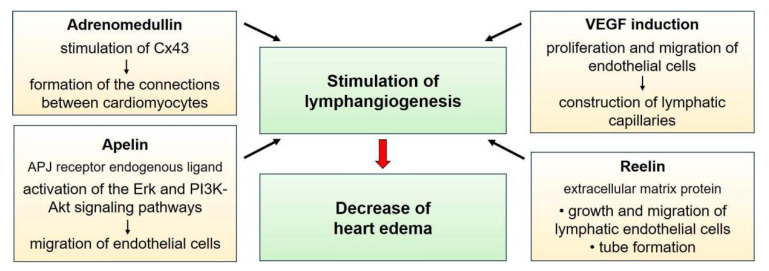
Therapeutic approaches for stimulation of lymphangiogenesis in the treatment of myocardial edema.

Considering the pathogenesis of myocardial edema, in which the main role is played by disruption of the lymphatic system of the heart, one of the most recent approaches is stimulation of lymphangiogenesis. Many studies in laboratory animals have shown that stimulation of lymphangiogenesis leads to a decrease in edema [82]. It improves cardiac function and slows the progression of myocardial fibrosis in the development of heart failure after myocardial infarction [18], suggesting that the lymphatic system may be an important target for controlling local myocardial edema. Therapeutic approaches aimed at stimulating lymphangiogenesis are presented in Figure 3.

Akt, protein kinase B; APJ, apelin receptor; Cx43, connexin 43; Erk, extracellular signal-regulated kinase; PI3K, phosphatidylinositol 3-kinase; VEGF, vascular endothelial growth factor

##### VEGF Induction

An important role in the process of lymphangiogenesis belongs to the most studied vascular endothelial growth factors (VEGFs), glycoproteins VEGF-C and VEGF-D [18,83]. Studies have shown that their levels increase during the first weeks after myocardial infarction. Some studies even indicate that low VEGF-C levels correlate with a worse prognosis in patients with coronary artery disease [84,85]. It is likely that insufficient production of these factors and impaired lymphangiogenesis aggravate myocardial remodeling [84]. Factors VEGF-C and VEGF-D are involved in the proliferation and migration of endothelial cells, as well as in the construction of lymphatic capillaries. These growth factors bind to two types of receptors: VEGFR3 and VEGFR2. Acting on VEGFR3 of lymphatic endothelial cells (LECs) through activation of the PI3K/AKT, MAPK/ERK, and MAPK/JNK signaling pathways [86], the growth factor stimulates lymphangiogenesis, and when binding to VEGFR2, angiogenesis is activated. In addition, VEGFA has been identified, acting through VEGFR1 and VEGFR2, as an angiogenic factor. VEGFA has been shown to promote LEC proliferation and migration as well as lymphangiogenesis through VEGFR1/2 expressed by LECs or through the recruitment of VEGFR1+ bone marrow-derived macrophages that secrete lymphangiogenic growth factors [87,88,89]. Despite such a promising approach, the issue remains controversial, as studies show conflicting results. A number of researchers have demonstrated that the introduction of factors improves cardiac function in mice and rats [90,91]. However, another study showed that turning off the genes of vascular growth factors or their receptors did not affect the deterioration of the pumping function of the heart after myocardial infarction [92]. In addition, these growth factors, by activating lymphangiogenesis, have been shown to stimulate metastasis of some tumors [93].

Various types of growth factor delivery to the myocardium have been proposed. Thus, gene delivery can be carried out using adenoviruses directly into the myocardium [90]. Researchers have proposed delivering VEGF-C plasmids into the myocardium of patients with coronary heart disease [18]. In addition, administration of soluble VEGFR3 also improves cardiac function by reducing T-cell infiltration [83,90].

##### Adrenomedullin

Lymphangiogenesis in the myocardium can also be stimulated by adrenomedullin (AM), a peptide produced in the adrenal glands, kidneys, heart, lungs, and adipose tissue. In the heart, adrenomedullin is produced in the myocardium of the ventricles and atria [94]. Adrenomedullin is a vasodilator peptide that acts through binding to the G-protein-coupled calcitonin receptor-like receptor (CRLR). It is a cardioprotective lymphangiocrine factor that improves cardiac function [95]. AM is formed by cleavage from the full-length proadrenomedullin (ProAM) precursor protein during its post-translational processing. To date, no beneficial biological effects of ProAM have been identified, but the use of AM leads to improved cardiac function [96]. As a result of adrenomedullin binding to the receptor heterodimers are formed with a member of the receptor activity-modifying proteins (RAMPs) family [97]. In mouse models with genetic loss of AM, CRLR, and RAMP proteins, it was shown that the CRLR-RAMP signaling mechanism through activation of the cAMP/PKA and PI3K/Akt signaling pathways plays an important role in the prevention of lymphatic edema [98]. Adrenomedullin is actively secreted by vascular endothelial cells. It is believed that adrenomedullin plays an important role in the formation of connections between cardiomyocytes, acting on Cx43. Thus, from experimental data on mice with overexpression of the adrenomedullin gene, it was possible to establish that it reduces swelling in the peri-infarction zone after 15–21 days and increases the number of lymphatic vessels in the heart (the number of vessels was determined using the LYVE-1 marker). In addition, the size of the vessels increased. In males, edema decreased faster. At the same time, in mice within 10 days after myocardial infarction, cardiac functions improved and the ejection fraction increased. Mice overexpressing adrenomedullin had higher levels of Cx43 in endothelial cells of the peri-infarct zone [94].

##### Apelin

Another substance that stimulates lymphangiogenesis is the protein apelin. Apelin, acting on the G-protein-coupled APJ receptor (the apelin receptor), stimulates the growth of blood and lymphatic vessels. Apelin receptors are localized to BECs (blood endothelial cells), LECs, SMCs (smooth muscle cells), and cardiomyocytes [99]. Stimulation of the receptors leads to activation of the Erk and PI3K-Akt signaling pathways. Apelin stimulates the migration of endothelial cells and reduces their apoptosis. It is involved in the development of the vascular system of the embryo, and also plays an important role in the proliferation of blood vessels in many malignant tumors. Research results show that apelin also stimulates the growth of lymphatic vessels in the tumor, and thus, promotes metastasis [99,100]. It was shown that apelin promotes the proliferation of lymphatic vessels in mice [99]. When the apelin gene was turned off in mice, lymphatic vessels dilated, their permeability increased, lymphatic drainage was impaired, and inflammation in the myocardial tissue intensified [83]. In turn, with overexpression of the gene, edema decreased and myocardial contractility increased [82]. Apelin has been shown to normalize lymphangiogenesis after ischemia and reduce the number of dilated vessels. Apelin appears to help reduce the release of atrial natriuretic peptide (ANP) and brain natriuretic peptide (BNP), which are involved in the development of myocardial fibrosis. It also acts on sphingosine kinase 1 and reduces fibroblast activity and the release of proinflammatory cytokines [99]. In addition, apelin may have beneficial effects in stem cell-based therapy. Thus, animal studies showed that its overexpression during mesenchymal stem cell (MSC) transplantation had a cardioprotective effect by activating AMP-activated protein kinase (AMPK) signaling [101].

##### Reelin

The protein reelin, an extracellular matrix protein, also has the potential to promote lymphangiogenesis. It is secreted by endothelial cells and promotes the maturation of cardiomyocytes in mouse embryos. Reelin expression is high after myocardial infarction in newly formed lymphatic capillaries. When the reelin gene was turned off in rat pups, a disturbance in the proliferation of cardiomyocytes after MI and their increased apoptosis was observed [82]. Reelin plays an important role in the growth, migration, and formation of lymphatic vessels. This protein acts in an autocrine fashion. LEC-derived reelin promotes lymphatic vascular development and lymphatic advancement through the production of monocyte chemotactic protein 1 (MCP-1) and subsequent recruitment of SMCs, which also stimulates the release of reelin from LECs. Further, reelin binds to its receptor integrin-β1, which regulates the function of cardiomyocytes and has a cardioprotective effect [102,103]. This fact is confirmed by studies in which exogenous delivery of reelin to mice after myocardial infarction helped to reduce apoptosis and fibrosis of cardiomyocytes [104].

#### 5.2.2. Other Therapy Approaches

It has been shown that statins (Hydroxymethylglutaryl-CoA (HMG-CoA)) reductase inhibitors), namely, atorvastatin in certain dosages, help to reduce the severity of myocardial edema [105]. Some studies indicate a beneficial effect of sodium–glucose cotransporter (SGLT) inhibitors in the treatment of chronic heart failure. These drugs (such as dapagliflozin, empagliflozin, etc.) are used for type 2 diabetes mellitus, as they interfere with the reabsorption of glucose in the renal tubules and reduce blood glucose levels. The drug empagliflozin has effects on the cardiovascular system: it reduces the incidence of cardiovascular complications in patients with diabetes and appears to have a cardioprotective effect. It reduces the extracellular fluid content in the myocardium and improves heart function [106]. In mice with induced diabetes mellitus and cardiomyopathy, when taking empagliflozin, the volume of fluid in the myocardium decreased. This may be explained by the fact that empagliflozin reduces the expression of aquaporins 1, 3, and 4 of cardiac endothelial cells. In mice with diabetes and cardiomyopathy, the levels of these aquaporins increased [107].

Thrombin is believed to increase the permeability of cardiac capillary endothelium. By phosphorylating tyrosine, it activates the MAPK pathway and disrupts the VE-cadherin complex. Thus, thrombin reduces the strength of adhesive contacts between endothelial cells. Based on this, aprotinin, a serine protease inhibitor that prevents the activation of thrombin in the blood (an antifibrinolytic agent), may be effective against myocardial edema. When aprotinin was administered to pigs after MI, the content of cadherins and catenins in the myocardium increased. Aprotinin prevented the degradation of adhesive contacts. It reduced the destruction of beta and gamma catenins associated with cadherins. A fluorescent method confirmed a higher content of cadherins and catenins, as well as junctions in the myocardium of pigs treated with aprotinin. In addition, it inactivates the p38 MAPK pathway associated with osmotic stress [25,108].

## 6. Conclusions

Myocardial edema often reflects acute cardiac disease. The etiology of edema is usually associated with ischemia or inflammation, and may therefore, be associated with chest pain or heart failure. Myocardial edema develops as a result of an imbalance between fluid filtration through the vascular wall and its removal through lymphatic capillaries from the myocardial interstitium and is considered a diagnostic marker of myocardial damage in vivo. Myocardial edema is present in a large number of acute and chronic cardiac pathologies: ischemia, myocardial infarction, ischemia/reperfusion, myocarditis, cardiac sarcoidosis, cardiomyopathy, sepsis, pulmonary hypertension, arterial hypertension, heart transplantation, and cardioplegic arrest.

Previously, edema could not be used as a diagnostic marker, since even histological methods could not provide reliable qualitative and, especially, quantitative data on its presence, extent, and location. T2-weighted cardiac MR images now allow visualization of edema, providing useful additional information in a variety of clinical situations where acute tissue injury is suspected. When combined with scar tissue visualization on T1-weighted MR images, reversible damage can be distinguished from irreversible damage.

The exact mechanisms of how edema affects function, long-term tissue composition, and electrical stability remain to be studied. Increased stiffness of the swelled myocardium significantly affects diastolic and systolic function, and altered fluid composition affects intramyocardial conduction and, therefore, the possibility of arrhythmia. Thus, myocardial edema is an important diagnostic target for assessing the severity and extent of tissue damage in vivo, and modeling of edema on two-dimensional and three-dimensional cell constructs will allow, without invasive methods, to advance the study of the mechanistic, morphological, and molecular changes.
